# The complete mitochondrial genome sequence of *Crenidorsum turpiniae* (Hemiptera: Aleyrodidae)

**DOI:** 10.1080/23802359.2020.1842264

**Published:** 2020-12-24

**Authors:** Shuai Li, Wei-Rui Wang, Yu-Feng Zhou, Li-Kun Zhong, Yan Jiang, Ze-Hong Meng

**Affiliations:** a Guizhou Tea Research Institute, Guizhou Academy of Agricultural Science, Guiyang, China; b College of Tea Science, Guizhou University, Guiyang, China; c Guizhou Institute of Biotechnology, Guizhou Academy of Agricultural Science, Guiyang, China; d Guizhou Provincial Key Laboratory for Agricultural Pest Management of the Mountainous Region, Institute of Entomology, Guizhou University, Guiyang, China

**Keywords:** Mitogenome, whitefly, tea pest, molecular phylogeny

## Abstract

We determined the complete mitochondrial genomes of *Crenidorsum turpiniae,* a new record whitefly pest on tea-tree. The mitogenome of *C. turpiniae* is 15,427 bp long and consists of 13 protein-coding genes, 22 tRNA genes, two rRNA genes and a putative control region (GenBank: MN934936). The whole base composition of the heavy strand for A, C, G and T is 30%, 12.24%, 15.82% and 41.87%, respectively, with an AT bias (-16%). All PCGs use ATN as start codon (N, any nucleotide), except for NAD6 uses TTG. Most of the PCGs use TAA as a stop codon. The length of 16SrRNA and 12SrRNA gene are 1277 bp and 768 bp, respectively. Phylogenetic analysis indicated that *C. turpiniae* and *Tetraleurodes acaciae* had a closer genetic relationship.

*Crenidorsum turpiniae,* a new record whitefly pest of tea tree, which took place frequently in Meitan County of Guizhou Province in China, belongs to Aleyrodidae of Hemiptera. Nymphs prefer to feed on the underside of leaves using piercing-sucking mouthparts, which cause dark mildew in tea plants and seriously impact tea quality and yield. Adults like to inhabit together at the back of tender tea leaves, but can lay eggs at the whole plants (Meng et al. 2017).

Specimens of *C. turpiniae* were collected from a tea garden at Meitan County (27°44′21.89″N, 107°32′23.56″E) of Guizhou Province in China, in October 2018. We extracted genomic DNA from adults of *C. turpiniae* via the rapid extraction kit for genomic DNA from tissue cells (Aidlab Biotechnologies Co., Ltd). The puparium and adult specimens were deposited in Guizhou Tea Research Institute, Guizhou Academy of Agricultural Science, Guiyang, China, with the voucher specimen number of ET20 and EU21, for further study. The sequencing results were assembled and annotated using DNAstar, analyzed and adjusted them manually, and finally obtained the complete mitochondrial sequence – 37 typical invertebrate mitochondrial genes (13 protein-coding genes (PCGs), 22 tRNAs, and 2 rRNAs) and the A + T-rich region (D-loop). Annotated sequence of *C. turpiniae* mitogenome was submitted to GenBank with accession number MN934936.

The complete mitogenome of *C. turpiniae* is 15,427 bp. The nucleotide composition is AT-biased (−16%), with the respectively proportion as follow: A = 30% (4637), C = 12.24%(1889), G = 15.82%(2441) and T = 41.87%(6460). In the mt genome of *C. turpiniae*, a total of 30 bp overlaps have been found at 12 gene junctions and 12 small non-coding portions (intergenic spacers), ranging in size from 1 to 20 bp, and a large 1063 bp non-coding region (A + T-rich region) was identified also.

The PCGs, ranging in size from 148 bp(ATP8) to 1657 bp(ND5), with a whole length of 10,890 bp. All PCGs use ATN as start codon (N, any nucleotide), except for NAD6 use TTG. The COX1 gene use TAG as a stop codon, for the same time three PCGs (COX2, NAD5 and ATP8) use single T as a stop codon, all the rests use TAA as a stop codon. The tRNAs, ranging from 56 (Ser) to 70 (Lys and Glu) bp, similar to other aleyrodids (Chen et al. 2016; Wang et al. 2016). All tRNA genes have the conserved triple nucleotides recognizing corresponding codon. The length of 16SrRNA and 12SrRNA gene are 1277 bp and 768 bp, respectively.

For understanding the evolutionary relationship of *C. turpiniae* in Aleyrodidae, the phylogenetic tree was constructed using MrBayes 3.2.0 (Ronquist et al. 2012) based on the nucleotide sequence of 13 PCGs of *C. turpiniae* and other 11 Aleyrodinae species (Figure 1). We found that *C. turpiniae* and *Tetraleurodes acaciae* were clustered into one clade, and closely with *Aleurocanthus camelliae* and *Aleurocanthus siniferus* in relationship. The molecular data of *C. turpiniae* in this study provided important data in evolutionary analysis of Aleyrodidae phylogeny.

**Figure 1.**
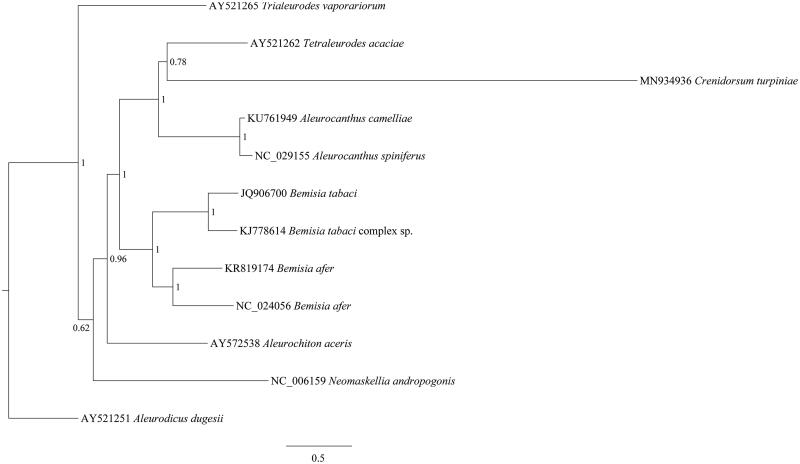
Phylogenetic tree of 12 species of Aleyrodidae (constructed by MrBayes 3.2.0 under the GTR + G + I model, run for 1,000,000 generations with a sampling frequency of 1000 generations).

## Data Availability

The sequence data generated in this study were openly available in GenBank (https://www.ncbi.nlm.nih.gov/nuccore/MN934936).
